# Enhancing the anti-angiogenic action of histone deacetylase inhibitors

**DOI:** 10.1186/1476-4598-6-68

**Published:** 2007-10-25

**Authors:** Selena Kuljaca, Tao Liu, Andrew EL Tee, Michelle Haber, Murray D Norris, Tanya Dwarte, Glenn M Marshall

**Affiliations:** 1 The Children's Cancer Institute Australia for Medical Research, The University of New South Wales, Sydney, NSW 2031, Australia; 2 The Centre for Children's Cancer and Blood Disorders, Sydney Children's Hospital, Randwick, Sydney, NSW 2031, Australia

## Abstract

**Background:**

Histone deacetylase inhibitors (HDACIs) have many effects on cancer cells, such as growth inhibition, induction of cell death, differentiation, and anti-angiogenesis, all with a wide therapeutic index. However, clinical trials demonstrate that HDACIs are more likely to be effective when used in combination with other anticancer agents. Moreover, the molecular basis for the anti-cancer action of HDACIs is still unknown. In this study, we compared different combinations of HDACIs and anti-cancer agents with anti-angiogenic effects, and analysed their mechanism of action.

**Results:**

Trichostatin A (TSA) and α-interferon (IFNα) were the most effective combination across a range of different cancer cell lines, while normal non-malignant cells did not respond in the same manner to the combination therapy. There was a close correlation between absence of basal p21^WAF1 ^expression and response to TSA and IFNα treatment. Moreover, inhibition of p21^WAF1 ^expression in a p21^WAF1^-expressing breast cancer cell line by a specific siRNA increased the cytotoxic effects of TSA and IFNα. *In vitro *assays of endothelial cell function showed that TSA and IFNα decreased endothelial cell migration, invasion, and capillary tubule formation, without affecting endothelial cell viability. TSA and IFNα co-operatively inhibited gene expression of some pro-angiogenic factors: vascular endothelial growth factor, hypoxia-inducible factor 1α and matrix metalloproteinase 9, in neuroblastoma cells under hypoxic conditions. Combination TSA and IFNα therapy markedly reduced tumour angiogenesis in neuroblastoma-bearing transgenic mice.

**Conclusion:**

Our results indicate that combination TSA and IFNα therapy has potent co-operative cytotoxic and anti-angiogenic activity. High basal p21^WAF1 ^expression appears to be acting as a resistance factor to the combination therapy.

## Background

Acetylation and deacetylation of histones by histone acetyltransferases and histone deacetylases (HDACs) alter chromatin structure and modulate transcriptional regulation (reviewed in [1-3]. Inhibitors of HDACs (HDACIs) are emerging as a new class of anticancer agents. HDACIs induce cancer cell differentiation, growth arrest, programmed cell death, and inhibit tumour-driven angiogenesis [1,3]. Clinical trials with HDACIs in cancer patients demonstrate that HDACI treatment leads to tumour regression and symptomatic improvement in some heavily pre-treated and multiply relapsed patients, with a surprisingly low side-effect profile [1,4]. However, a large proportion of the patients are not sensitive to the treatment, demonstrating the need to examine the effectiveness of HDACIs in combination with other anti-cancer agents.

Angiogenesis is vital for tumor progression and metastasis [5,6]. As anti-angiogenic therapy is generally less toxic and better tolerated than conventional cytotoxic chemotherapy, strategies which combine anti-angiogenic agents with other anti-cancer drugs have been the focus of current clinical trials to widen the therapeutic index. The interferons (IFNs) are a family of naturally occurring cytokines with anti-proliferative and anti-angiogenic effects [7,8]. Through inhibiting pro-angiogenic gene expression and acting directly on endothelial cells, α-interferon (IFNα) suppresses angiogenesis and tumour growth *in vitro *and *in vivo *[7,9]. Rapamycin and its derivatives also inhibit tumour cell proliferation and angiogenesis by acting on the mammalian target of rapamycin and suppressing the transcriptional activity of pro-angiogenic hypoxia-inducible factor 1α (HIF1α), (reviewed in [10]). While clinical trials with IFNα, rapamycin and its derivatives used as single agents have shown some effects, none of the drugs are effective alone in the majority of patients.

It has been reported that a combination therapy with the HDACI, valproate (VPA), and IFNα exerts synergistic anti-cancer effects in neuroblastoma BE(2)-C cells both *in vitro *and *in vivo *[11,12]. Here we evaluated the anticancer actions of combination therapy with HDACIs (Trichostatin A [TSA] or VPA) and anti-cancer agents with anti-angiogenic function (IFNα, rapamycin), and, sought to determine their mechanism of action.

## Results

### TSA and IFNα exerted co-operative cytotoxic effects in cancer cell lines from a range of different tissue origins

**Figure 1:**
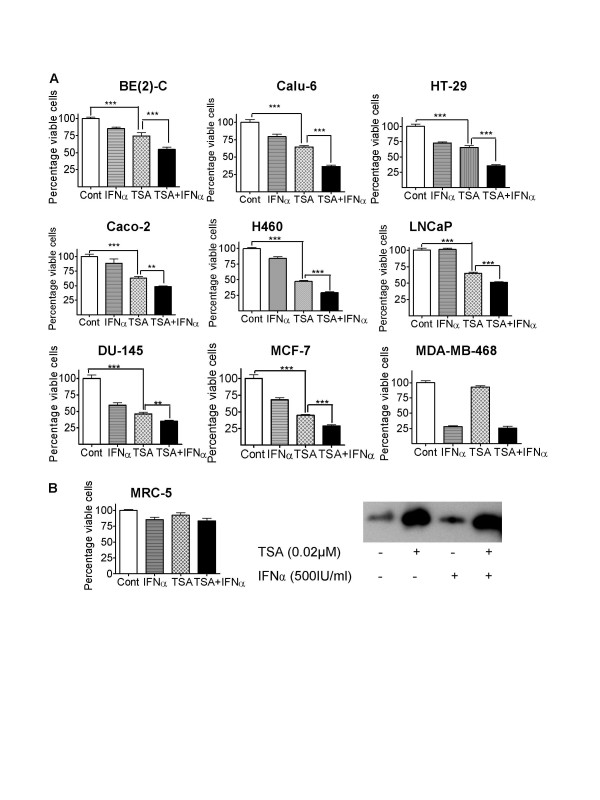
TSA and IFNα exerted co-operative cytotoxic effects in cancer cell lines from a range of different tissue origins, but not in normal non-malignant cells. **A. **Neuroblastoma [BE(2)-C], breast (MCF-7 and MDA-MB-468), lung (H460 and Calu-6), prostate (DU-145 and LNCaP), and colon (HT-29 and Caco-2) cancer cells were treated with control (Cont), 0.02 μM TSA and/or 500 IU/ml IFNα for 72 hours. Cell viability was examined using the Alamar blue assay, measured as optical density (OD) units of absorbance, and expressed as the absorbance of treated over control samples (ie., % viable cells). ** p < 0.01, *** p < 0.001. B. MRC-5 cells were treated with control, 0.02 μM TSA and/or 500 IU/ml IFNα for 72 hours, and cell viability was assessed as above. Moreover, histone protein was extracted and subject to immunoblot analysis with anti-acetylated histone H3 antibody, after 6 hour exposure to control, TSA and/or IFNα.

The combination of the HDACI, VPA, and IFNα demonstrated synergistic combinational anti-cancer effects in neuroblastoma BE(2)-C cells both *in vitro *and *in vivo *[11,12]. We investigated the synergistic anti-cancer effect of IFNα combined with other HDACIs, and, in cancer cell lines of other tissue origins. We treated breast, lung, colon and prostate cancer cells and MRC-5 normal non-malignant fibroblasts with control, 0.02 μM TSA and/or 500 IU/ml IFNα, and, then assessed for cell viability. As shown in Figure 1A, all of the cancer cell lines tested were sensitive to the cytotoxic effects of the combination, and there was a significant cooperative effect of TSA and IFNα in eight of the nine cell lines tested, with MDA-MB-468 as the only exception. MCF-7, Calu-6, H460, LNCaP, DU-145, HT-29, Caco-2 and BE(2)-C cells were all sensitive to TSA, generally less sensitive to IFNα, and significantly more sensitive to TSA and IFNα combined. MDA-MB-468 breast cancer cells were sensitive to IFNα but resistant to TSA, and no more sensitive to the combination than IFNα alone. When cell sensitivity to the combination treatment was calculated as a percentage of TSA alone (or IFNα alone in case of MDA-MB-468), BE(2)-C, HT-29 and Calu-6 were found to be the most sensitive (Figure 1A). Importantly, the normal non-malignant MRC-5 fibroblasts were resistant to the treatment of TSA alone, IFNα alone and TSA plus IFNα combination therapy (Figure 1B). Immunoblot analysis of acetylated histone H3 revealed that treatment with TSA alone or TSA plus IFNα for 6 hours induced drastic histone acetylation in the MRC-5 cells (Figure 1B).

### SAHA and IFNα exerted co-operative cytotoxic effects in cancer cell lines, but not in normal cells

**Figure 2:**
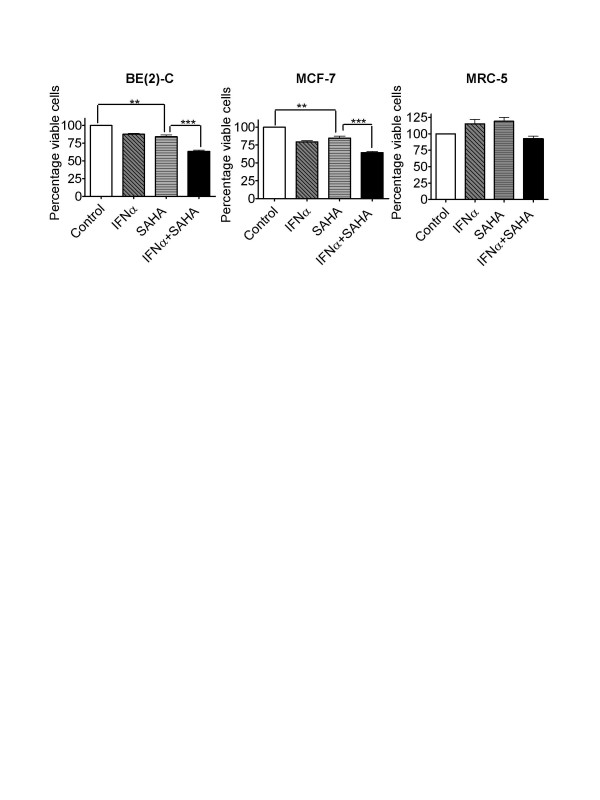
SAHA and IFNα exerted co-operative cytotoxic effects in cancer cell lines, but not in normal non-malignant cells. Neuroblastoma BE(2)-C, breast cancer MCF-7, and normal non-malignant lung MRC-5 fibroblasts were treated with control, 0.5 μM SAHA and/or 500 IU/ml IFNα for 72 hours. Cell viability was examined using the Alamar blue assay, measured as optical density (OD) units of absorbance, and expressed as the absorbance of treated over control samples (ie., % viable cells). * p < 0.05, ** p < 0.01, *** p < 0.001.

The HDACI, SAHA (vorinostat), is in clinical use for the treatment of cutaneous T-cell lymphoma. We, therefore, tested whether a combination of SAHA and INFα exerted co-operative anti-cancer effects. Neuroblastoma BE(2)-C, breast cancer MCF-7, and normal lung fibroblast MRC-5 cells were treated with control, 0.5 μM SAHA, 500 IU/ml INFα or SAHA plus INFα for 3 days. Alamar blue assays revealed that SAHA and INFα co-operatively reduced the viability of BE(2)-C and MCF-7 cells, although the magnitude was smaller than TSA and INFα. A combination of SAHA and INFα did not co-operatively reduce the viability of MRC-5 cells (Figure 2).

### The effects of other HDACIs and anti-cancer agents used in combination

**Figure 3:**
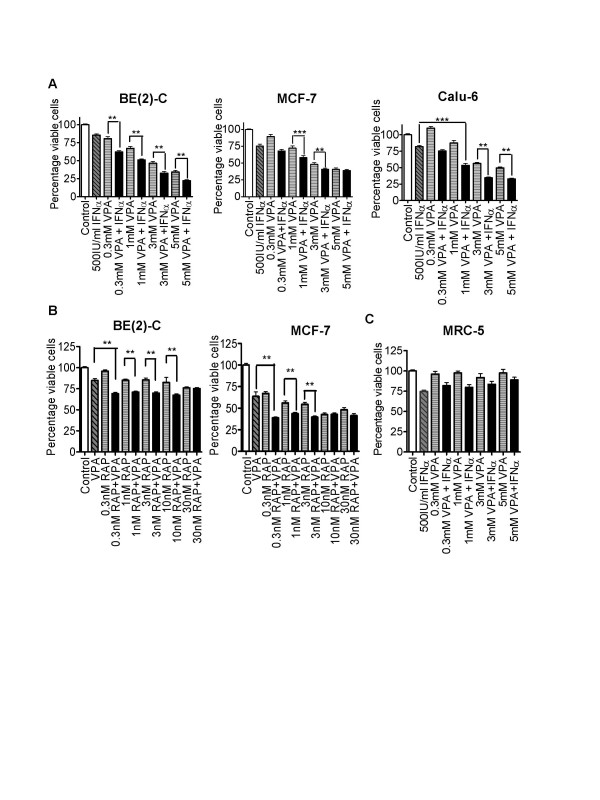
The cytotoxic effects of other HDACI combination therapies. A. Neuroblastoma [BE(2)-C], breast (MCF-7), and lung (Calu-6) cancer cell lines were treated with either control, 500 IU/ml IFNα and/or various dosages of VPA for 72 hours B. In separate experiments, BE(2)-C and MCF-7 cells were treated with control, 1 mM VPA and/or various dosages of rapamycin (RAP) for 72 hours. C. Non-malignant lung fibroblast (MRC-5) cells were treated with a range of VPA doses alone, or in combination with 500 IU/ml IFNα. Cell viability was examined by the Alamar blue assay, measured as optical density (OD) units of absorbance, and expressed as a percentage of absorbance for treated samples, over that for control samples (ie., % viable cells). ** p < 0.01, *** p < 0.001.

We compared the cytotoxicity of the TSA and IFNα combination (Figure 1A) with combinations of another HDACI VPA and IFNα (Figure 3A). The effect of VPA and IFNα combination therapy on cell viability was similar to TSA and IFNα for the BE(2)-C neuroblastoma cells. However, TSA and IFNα were more effective in MCF-7 and Calu-6 cells than VPA and IFNα. Similar to TSA and IFNα, VPA and IFNα did not show any co-operative cytotoxic effects on normal MRC-5 fibroblasts (Figure 3C). We next compared the cytoxicity of the TSA and IFNα combination (Figure 1A), the VPA and IFNα combination (Figure 3A) with VPA combined with another emerging anticancer agent with both cytotoxic and anti-angiogenic actions, rapamycin [10] in BE(2)-C and MCF-7 cells (Figure 3B). The VPA and rapamycin treatment had significant cytotoxic effects compared with VPA alone, but the magnitude of these effects was much smaller than VPA and IFNα or TSA and IFNα.

### Absence of p21^WAF1 ^expression correlated with sensitivity to TSA and IFNα combination therapy

**Figure 4:**
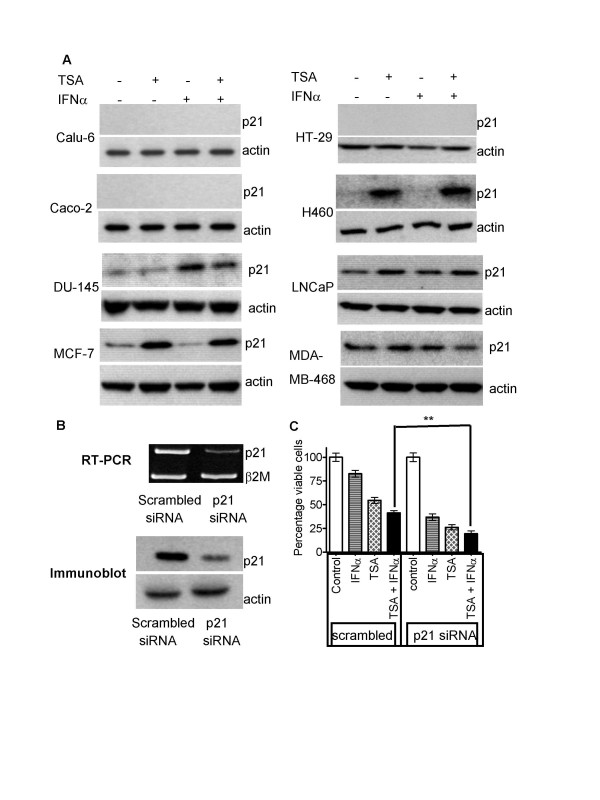
Absence of p21^WAF1 ^expression correlated with sensitivity to TSA and IFNα combination therapy. A. MCF-7, MDA-MB-468, H460, Calu-6, DU-145, LNCaP, HT-29 and Caco-2 cells were treated with control, 0.02 μM TSA, 500 IU/ml IFNα, or TSA and IFNα for 24 hours. Whole cell protein was extracted and subjected to immunoblot with an anti- p21^WAF1 ^antibody, and, an anti-actin antibody as a loading control. B. MCF-7 cells were transfected with control scrambled or p21^WAF1 ^siRNA for 8 hours, followed by treatment with control, 0.02 μM TSA and/or 500 IU/ml IFNα for 72 hours. The effect of the siRNAs on p21^WAF1 ^gene and protein expression was analysed by semi-quantitative RT-PCR with the house-keeping gene β-2-microglobulin (β2M) as a loading control or by immunoblot, with actin as a loading control. C. Cell viability was examined by the Alamar blue assay, measured as optical density (OD) units of absorbance, and expressed as percentage of absorbance for drug-treated samples over control-treated samples (% viable cells). ** p < 0.01.

Up-regulation of p21^WAF1 ^expression, and p21^WAF1^-induced cell cycle arrest, have been regarded as one of the main mechanisms through which HDACIs exert their anti-cancer effects[13]. We examined the role of p21^WAF1 ^in cancer cell sensitivity to the combination therapy. Immunoblot analysis of p21^WAF1 ^expression was carried out with protein extracted from the eight cell lines of breast, lung, prostate and colon origins (Figure 4A). p21^WAF1 ^was basally expressed in untreated H460, DU-145, LNCaP, MCF-7 and MDA-MB-468 cells, but not expressed in Calu-6, HT-29 and Caco-2 cells. Compared with control, TSA induced p21^WAF1 ^expression in H460, MCF-7 and LNCaP cells. IFNα up-regulated p21^WAF1 ^only in DU-145 cells, and, combination therapy increased p21^WAF1 ^in all four cell lines. p21^WAF1 ^protein was expressed but not altered by treatment with TSA and/or IFNα in MDA-MB-468 cells. Thus, the cancer cell lines which did not have basal expression of p21^WAF1 ^were generally more sensitive to the combination therapy than those cancer cells expressing p21^WAF1^. This correlation suggested expression of p21^WAF1 ^might render cancer cells insensitive to the combination therapy.

To determine the role of p21^WAF1 ^expression in cancer cell sensitivity to TSA and IFNα combination therapy, MCF-7 cells were transfected with control, scrambled siRNA or siRNA specifically targeting p21^WAF1^, and, then treated with control, TSA and/or IFNα. RT-PCR and immunoblot analysis revealed that p21^WAF1 ^mRNA and protein were knocked down by approximately 75% by the p21^WAF1 ^siRNA, compared with scrambled control (Figure 4B). The p21^WAF1 ^siRNA significantly increased the sensitivity of MCF-7 cells to TSA and IFNα alone, and, in combination, as measured by cell viability assays (p < 0.01) (Figure 4C).

### HDACI and IFNα co-operatively inhibit endothelial cell functions and pro-angiogenic gene expression in cancer cells in vitro

**Figure 5:**
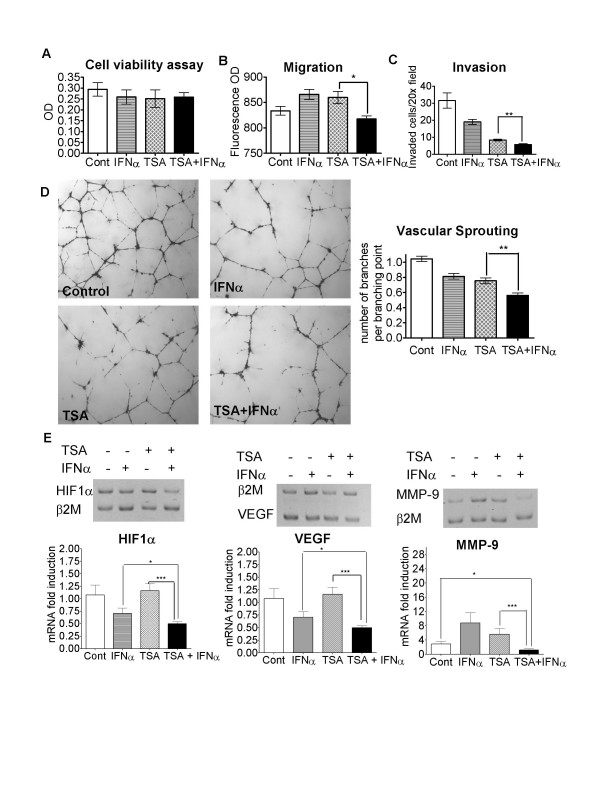
HDACI and IFNα co-operatively inhibit endothelial cell functions, and pro-angiogenic gene expression in cancer cells under hypoxic conditions *in vitro*. A. Human umbilical vein endothelial cells (HUVECs) were treated with control (Cont), 0.1 μM TSA and/or 500 IU/ml IFNα for 18 hours. Cell viability was evaluated with the Alamar blue assay. B. HUVECs were plated in BD Biosciences Fluroblok chambers and treated with control, 0.1 μM TSA and/or 500 IU/ml IFNα for 22 hours. Cells were stained with Cell Tracker Green CMFDA, migrated through chamber filters toward the chemo-attractant VEGF, and then quantified and expressed as optical density (OD) absorbance units. C. HUVECs were plated into BD BioCoat growth factor-reduced matrigel invasion chambers and treated with control, 0.1 μM TSA and/or 500 IU/ml IFNα for 18 hours. Cells which invaded through the Matrigel were fixed, stained with a Diff Quick staining kit, photographed and then quantified. D. HUVECs were plated onto growth factor-reduced Matrigel in 24 well plates and treated with control, 0.1 μM TSA and/or 500 IU/ml IFNα for 18 hours. Vascular sprouting was quantified by counting the numbers of complete branches per branching point. E. Neuroblastoma BE(2)-C cells were treated with control, 0.02 μM TSA and/or 500 IU/ml IFNα for 72 hours under hypoxic (1% O_2_) conditions. RNA was extracted and subjected to independent semi-competitive RT-PCR analyses using trans-intron PCR primers, together with primers for the house-keeping gene β-2 microglobulin (β2M). Representative gels for each gene at the 72 hour time point were shown, and fold induction of a target gene by treatment was calculated by ascribing the ratio between the level of expression of a target gene and that of β2M as 1.0 for control treated samples. * p < 0.05, ** p < 0.01, *** p < 0.001.

Since HDACIs [2,14] and IFNα [8,9] are known to suppress angiogenesis and tumour growth by acting directly on endothelial cells, we further investigated whether the combination of TSA and IFNα could inhibit endothelial cell function. To exclude the possibility that co-operative anti-angiogenic effects by TSA and IFNα were due to cytotoxicity, we first determined the optimal dosages of TSA and IFNα with Alamar blue cell viability assays. After treatment for 18 hours under normoxic or hypoxic conditions (1% O_2_), a combination of 0.1 μM TSA and 500 IU/ml IFNα was found to have no cytotoxicity on endothelial cells within 18 hours after treatment (Figure 5A). These doses were, therefore, used in all endothelial cell function studies. Surprisingly, TSA or IFNα alone stimulated endothelial cell migration toward the chemoattractant, vascular endothelial growth factor (VEGF) (Figure 5B). In contrast, the combination of TSA and IFNα suppressed endothelial cell migration under both hypoxic (Figure 5B) or normoxic conditions (data not shown). Compared with control, IFNα or TSA alone reduced endothelial cell invasion through Matrigel by 35% and 60%, respectively, whereas the combination of TSA and IFNα decreased cell invasion by 80%, under normoxic (data not shown) or hypoxic conditions (Figure 5C). Under normoxic conditions, compared with control, IFNα or TSA alone decreased the number of complete branches per branching point by 30% and 50%, respectively, while TSA and IFNα did not further decrease complete branches per branching point (data not shown). In contrast, under hypoxic conditions, the combination of TSA and IFNα decreased complete branches per branching point by 50%, while TSA or IFNα alone reduced the average numbers of complete branches from a branching point by only 25% (Figure 5D).

We next evaluated whether the combination of TSA [15,16] and IFNα [7,17] represses pro-angiogenic gene expression, as measured by RT-PCR, in neuroblastoma BE(2)-C cells. Compared with treatment with TSA or IFNα alone, the combination therapy significantly down-regulated gene expression of HIF1α, VEGF and MMP-9 under normoxic conditions at 72 hours after treatment, while no co-operative effects were observed on the expression of MMP-2, activin A, thrombospondin-1, von Hippel-Lindau protein and bFGF (data not shown). Suppression of HIF1α, VEGF and MMP-9 gene expression by TSA and IFNα was more significant, when compared with TSA or IFNα alone, under hypoxic conditions (Figure 5E). In the case of HIF1α and VEGF, IFNα alone repressed gene expression, however, the combination still had a more significant repressive effect, compared with IFNα alone (p < 0.05). Although MMP-9 gene expression was stimulated by IFNα and TSA alone, the combination suppressed its expression, when compared with control-treated samples (p < 0.05).

### TSA and IFNα co-operatively suppress tumour-driven angiogenesis in neuroblastoma-bearingN-Myc transgenic mice

**Figure 6:**
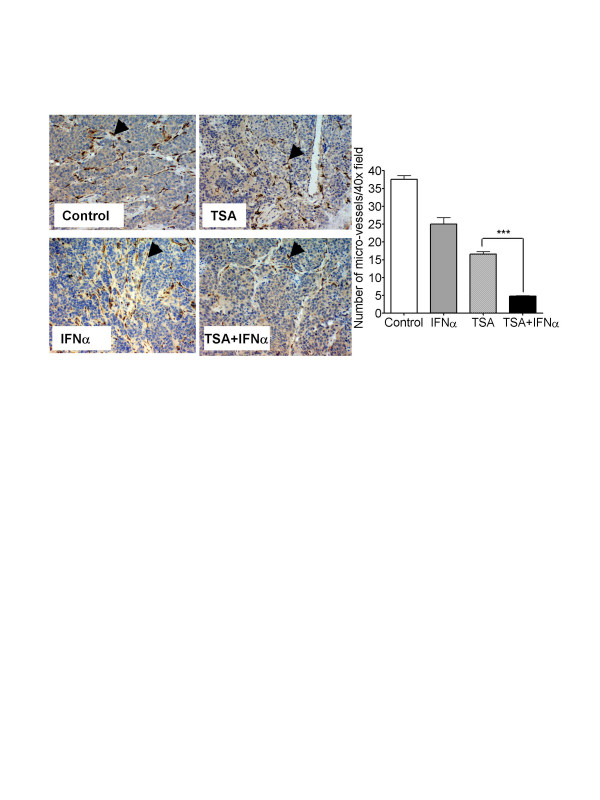
TSA and IFNα co-operatively suppress tumour-driven angiogenesis in neuroblastoma bearing transgenic MYCN mice. A. Photomicrographs of neuroblastoma tumour tissue sections from homozygous MYCN transgenic mice treated with either control, TSA, IFNα, or TSA and IFNα, which were subject to immunohistochemical studies using an anti-PECAM-1 antibody. Arrows indicate PECAM-1 positive microvessels (brown colored). B. Quantitation of the number of PECAM positive microvessels per 40× high power field in neuroblastoma tumour cross-sections. *** p < 0.001.

Lastly, we tested whether the combination of TSA and IFNα could co-operatively inhibit tumor-driven angiogenesis *in vivo*. Abdominal neuroblastoma first became palpable in 100% of homozygote N-Myc transgenic mice at 4 weeks of age [18]. Cohorts of five homozygous MYCN transgenic mice at four weeks of age, were treated with control, IFNα, TSA, or TSA and IFNα for one week after abdominal tumors were first palpable. After mice were sacrificed, tumour volume was measured, and microvasculature assessed by immunohistochemical staining for platelet endothelial cell adhesion molecule 1 (PECAM-1) expression (Figure 6). When tumour volume was analysed, TSA alone suppressed tumour progression by 87%, while IFNα alone reduced tumour volume by about 36%, compared with control treated mice. The combination of TSA and IFNα reduced tumour volume by more than 92%, although this was not statistically significant compared with TSA treatment alone. When tumour micro-vasculature was assessed by PECAM-1 staining, the use of TSA or IFNα alone, decreased micro-vasculature formation by 32% and 53%, respectively. However, the combination of TSA and IFNα exerted co-operative anti-angiogenic effects, reducing micro-vasculature by almost 90% (Figure 6)

## Discussion

HDACIs have shown great promise in clinical trials in cancer patients. However, a majority of patients have been insensitive to the treatment. In this study, we found that the combination of IFNα with the HDACI TSA induced co-operative cytotoxic effects in almost all cancer cell lines of diverse tissue types, and demonstrated little cytoxicity in normal non-malignant cells. The combination of IFNα with the HDACI SAHA, already in clinical use, also exerted co-operative anti-cancer effects, with little effect on normal cells. The combination of IFNα with another HDACI, VPA, was less effective than IFNα and TSA, but more effective than VPA and rapamycin. These results suggest that HDACI and IFNα combination therapy may be an effective anti-cancer strategy for future clinical trials.

Our data identified p21^WAF1 ^expression as a key factor responsible for cancer cell resistance to the cytotoxic effects of combination HDACI and IFNα therapy. While IFNα can both induce or suppress p21^WAF1 ^gene transcription in different cells [19], it is the most common transcriptional target of HDACIs (reviewed in [2]). Previous literature suggested that up-regulation of p21^WAF1 ^by HDACIs may mediate HDACI-induced cell cycle arrest and growth inhibition [13]. However, recent publications have cast doubt on the role of p21^WAF1 ^in the action of HDACIs, and, conversely demonstrated that inducible p21^WAF1 ^reduced HDACI-induced cell death [20-24]. Our data suggests p21^WAF1 ^expression in some cancer cells acts as a resistance factor for the cytotoxic effects of TSA and IFNα combination therapy.

The individual effects of HDACIs and IFNα on angiogenesis predict a co-operative therapeutic role in blocking tumour angiogenesis. Expression of HDACs is often up-regulated under angiogenic stimuli such as hypoxia in cancer cells, and HDACIs can suppress HIF1α expression and its down-stream targets, including VEGF[25]. HDACIs have been recently demonstrated to inhibit endothelial cell migration, invasion, vascular sprouting *in vitro*, and vasculature formation in animal models of cancer [14,16,26]. IFNα can repress VEGF and MMP-9 gene expression, endothelial cell functions, and, inhibit tumour-driven angiogenesis *in vivo *[9,27]. In our endothelial cell migration experiments, we found in contrast, that either TSA or IFNα alone stimulated migration. We cannot fully explain the discrepancy between our data and previously published migration assays [14], however, this may be due to different characteristics of the migration chamber used. Importantly, the combination of HDACI and IFNα suppressed all endothelial cell functions, indicating a possible role for this drug combination as a therapy for cancer patients at the point of minimal residual disease.

## Conclusion

In summary, we have found that the combination of HDACIs, TSA, SAHA and VPA, with IFNα have significant cytotoxic effects on a wide variety of cancer cells, with little toxicity to normal non-malignant cells. Inhibition of p21^WAF1 ^expression sensitizes p21^WAF1^-expressing cancer cells to the combination therapy. Furthermore, HDACI and IFNα co-operatively suppress pro-angiogenic gene expression in cancer cells, multiple endothelial cell functions *in vitro*, and tumour-driven vasculature formation *in vivo*. Our results provide a basis for further *in vivo *studies and eventual clinical trials using the combination of HDACIs and IFNα.

## Methods

Cell culture and reagentsThe neuroblastoma cell line, BE(2)-C, was generously supplied by Dr J Biedler (Memorial Sloan-Kettering Cancer Center, NY, USA). Breast (MCF-7 and MDA-MB-468), lung (Calu-6 and H460), prostate (DU-145 and LNCaP), and, colon (HT-29 and Caco-2) cancer cells were purchased from American Type Culture Collection (Manassas, VA, USA). All cell lines were cultured in Dulbecco's modified Eagle's medium supplemented with 10% fetal calf serum, with the exception of H460 and LNCaP, which were cultured in Roswell Park Memorial Institute Medium, supplemented with 10% fetal calf serum. All cell lines were maintained in a humidified incubator at 37°C and 5% CO_2 _in air.

TSA (Sigma, St. Luis, MO, USA) was dissolved in ethanol, and SAHA (BioVision, Mountain View, CA) in dimethylsulfoxide (Sigma). IFNα (Sigma) was diluted in serum free cell culture medium and aliquoted as a stock solution of 100 000 units/ml. For studies in animals, TSA was dissolved in dimethyl sulfoxide (Sigma) and further diluted with saline solution to give the final concentration of 30% dimethyl sulfoxide and 1 mg/ml TSA.

### Endothelial cell culture

Human umbilical vein endothelial cells (HUVECs) were a gift from Dr K MacKenzie (Children's Cancer Institute Australia, Sydney, Australia). HUVECs were maintained in 0.1% gelatin coated tissue culture flasks or wells with medium 199 (Invitrogen, Carlsbad, CA, USA) supplemented with 20% fetal bovine serum, 5% human serum (Sigma), 10 U/ml heparin (Pharmacia & Upjohn, Peapack, NJ, USA), 5 ng/ml basic fibroblast growth factor (bFGF) (Sigma) and 20 ug/ml endothelial growth factor (Roche, Mannheim, Germany). Only passages 5 and 6 were used in the experiments. Hypoxic conditions were maintained in a chamber filled with 1% oxygen.

### Alamar blue cell viability assay

After plating in 96 well plates, cells were allowed to attach for 24 hours, followed by treatment with various drugs for 72 hours. Before the end of treatment, cells were incubated with Alamar blue (Invitrogen) for 5 hours, and plates were then read on a micro-plate reader at 570/595 nm. Relative cell viability was calculated according to the readings and expressed as optical density (OD) absorbance units.

### Immunoblot analysis

Twenty four hours after treatment with control, TSA and/or IFNα, protein was extracted from whole cells, separated by electrophoresis, and transferred onto nitrocellulose membrane. Membranes were incubated with mouse anti-human p21^WAF1 ^antibodies (Santa Cruz Biotechnologies, Santa Cruz, CA, USA) (1:1000), followed by goat anti-mouse antibody (1:2000) conjugated with horseradish peroxidase. Chemiluminescent detection was performed using SuperSignal reagents (Pierce). Membranes were then re-probed with an anti-β-actin antibody (Pierce), as a loading control.

siRNA transfectionMCF7 cells were transfected with a validated scrambled siRNA or siRNA specifically targeting p21^WAF1 ^(SmartPool siRNA CDKN1A, Dharmacon Research, Lafayete, CO) with Lipofectamine 2000 transfection reagent (Invitrogen) according to the manufacturer's recommendation. Cells were lysed, and RNA or protein extracted 24 hours later for Reverse Transcription-polymerase Chain Reaction (RT-PCR) or immunoblot analysis of siRNA transfection efficacy.

### Semi-quantitative competitive RT-PCR

Semi-quantitative competitive RT-PCR was carried out as described previously [28] to analyse siRNA transfection efficiency in MCF-7 cells and the effect of TSA and/or IFNα treatment on angiogenic gene expression in BE(2)-C cells. Specific primers used for PCR were as follows: 5'-CAGCAGAGGAAGACCATGTG-3' and 5'-GGCGTTTGGAGTGGTAGAAA-3' for p21^WAF1^; 5'-TTACAGCAGCCAGACGATCA-3' and 5'-ATTGCCCCAGCAGTCTACAT-3' for HIF1α; 5'-CCTTGCTGCTCTACCTCCAC-3' and 5'-ATGATTCTGCCCTCCTCCTT-3' for vascular endothelial growth factor (VEGF); 5'-TTCCCTGGAGACCTGAGAAC-3' and 5'-AGGGACAGTTGCTTCTGGAG-3' for metalloproteinase-9 (MMP-9); 5'-ACCCCCACTGAAAAAGATGA-3' and 5'-ATCTTCAAACCTCCATGATG-3' for β2-microglobulin (β2M).

### Endothelial cell migration assay

HUVEC migration towards the chemo-attractant, VEGF (Sigma), was tested using a BD Biosciences Fluroblok (Becton Dickinson) endothelial cell migration system according to the manufacture's guidelines. Cells were labeled with 1 μM Cell Tracker Green CMFDA fluorescence solution (Invitrogen) for 30 minutes, and migrated through filters into 24 well plates. Thereafter the plate was read with a Fluroscence plate reader at 492/517 nm. The relative cell number was calculated according to the readings and expressed as optical density (OD) absorbance units.

### Endothelial cell invasion assay

HUVEC invasion through matrigel towards the chemo-attractant, VEGF, was investigated using BD BioCoat, growth factor-reduced Matrigel, endothelial cell invasion chambers (Becton Dickinson), according to the manufacturer's guidelines. Endothelial cells which invaded through the matrigel to the other side of the inserts, were fixed and stained with Diff Quick staining kit (Baxter) and photographed. The number of cells per 20× objective field was counted under an inverted microscope.

### Vascular sprouting (capillary tubule formation) assay

The vascular sprouting assays were performed on 24 well plates coated with 250 μl of polymerized, growth factor-reduced Matrigel matrix (Becton Dickinson) per well. HUVECs were plated on Matrigel and treated with control, TSA and/or IFNα for 18 hours. Quantification of vascular sprouting was determined by counting the number of complete branches per branching point.

### Animal model studies

As soon as tumors were confirmed by abdominal palpation, MYCN homozygous transgenic mice [18], were randomized to four groups (n = 5/group) and injected intraperitoneally daily for 7 days with control, TSA at 20 mg/kg of body weight, mouse IFNα at 1 × 10^6 ^IU/kg body weight, or TSA and IFNα. Mice were sacrificed at the end of the week of treatment. Tumors were then removed, formalin-fixed and paraffin-embedded. All studies involving animals were approved by the animal care and ethics committee of the University of New South Wales, Sydney, Australia.

### Immunohistochemical studies

Mouse tissue sections were incubated with goat anti-platelet endothelial cell adhesion molecule 1 (PECAM-1) antibody (1:500) (Santa Cruz Biotechnology), followed by incubation with biotinylated rabbit, anti-goat antibody (1:500) and streptavidin-horseradish peroxidase. Endothelial cells were visualised with 3,3'-diaminobenzidine solution, and micro-vessels were quantified as described previously [29].

### Statistical analyses

All data for statistical analyses were presented as mean ±standard error. Differences were analyzed for significance using ANOVA among groups. A probability value of 0.05 or less was considered significant.

## Abbreviations

bFGF: basic fibroblast growth factor; Cont: control; HDAC: histone deacetylase; HDACI: histone deacetylase inhibitor; HIF1α: hypoxia-inducible factor 1α; HUVEC: human umbilical vein endothelial cells; IFNα: α-interferon; MMP-9: matrix metalloproteinase 9; OD: optical density; PECAM-1: platelet endothelial cell adhesion molecule 1; TSA: Trichostatin A; RAP: rapamycin; VEGF: vascular endothelial growth factor; VPA: valproate

## Competing interests

The author(s) declare that they have no competing interests.

## Authors' contributions

SK, TL, AT and TD performed experiments and analysed data. GMM, MH and MN designed experiments. TL and GMM analysed data and wrote the manuscript. All authors have read and approved the final version of the manuscript.
